# Stem Cells for the Treatment of Neurodegenerative Diseases

**DOI:** 10.3390/molecules15106743

**Published:** 2010-09-27

**Authors:** Yong-Ping Wu, Wei-Shan Chen, Chong Teng, Ning Zhang

**Affiliations:** Department of Orthopaedics, 2nd Affiliated Hospital, School of Medicine, Zhejiang University, #88 Jiefang Road, Hangzhou, 310009, China

**Keywords:** stem cell, neurodegenerative diseases, treatment

## Abstract

Neurodegenerative diseases are characterized by neurodegenerative changes or apoptosis of neurons involved in networks, leading to permanent paralysis and loss of sensation below the site of the injury. Cell replacement therapy has provided the basis for the development of potentially powerful new therapeutic strategies for a broad spectrum of human neurological diseases. In recent years, neurons and glial cells have successfully been generated from stem cells, and extensive efforts by investigators to develop stem cell-based brain transplantation therapies have been carried out. We review here notable previously published experimental and preclinical studies involving stem cell-based cell for neurodegenerative diseases and discuss the future prospects for stem cell therapy of neurological disorders in the clinical setting. Steady and solid progress in stem cell research in both basic and preclinical settings should support the hope for development of stem cell-based cell therapies for neurological diseases.

## 1. Introduction

Neurodegenerative diseases, such as Parkinson’s disease (PD), stroke, Huntington’s disease (HD) and amyotrophic lateral sclerosis (ALS), are characterized by neurodegenerative changes or apoptosis of neurons involved in networks, leading to permanent paralysis and loss of sensation below the site of the injury [1]. Unfortunately, so far no successful treatment for neurodegenerative diseases has been developed. Cell replacement therapy and gene transfer to the diseased or injured brain have provided the basis for the development of potentially powerful new therapeutic strategies for a broad spectrum of human neurological diseases. Stem cells are capable of repairing injured nervous tissue by replacing damaged cells, neuroprotection or the creation of an environment conducive to regeneration by endogenous cells [2]. The transplantation of stem cells may provide effective treatments due to the self-renewing and multipotential nature of these cells, including delivery of therapeutic factors to provide trophic support or missing gene products, mobilization of endogenous stem cells and replacement of lost or dysfunctional cells. 

Meanwhile, human embryonic stem cells (ESCs) and adult stem cells have been coaxed into types of cells that repair neurodegenerative diseases insulation and replace nerve cells in neurodegenerative diseases [3]. The potency of these cells and the relative ease of isolating and expanding them are invaluable properties for clinical application. And some clinical trials have also been undertaken in neurodegenerative diseases [4,5]. Steady and solid progress in stem cell research in both basic and preclinical settings should support the hope for development of stem cell-based cell therapies for neurodegenerative diseases.

## 2. Stem Cell and Neurodegenerative Diseases

Here we review the scientific basis of stem cell therapies and discuss their prospects in Parkinson’s disease, Huntington's disease, Alzheimer's disease, Amyotrophic Lateral Sclerosis, stroke and spinal cord injury. In each of these neurodegenerative diseases, we describe the ways in which stem cells might be used to treat these conditions, discussing the prospects and problems of translating laboratory findings into clinically useful therapies.

### 2.1. Stem cells and Parkinson’s disease

Parkinson’s disease is a neurodegenerative disorder characterized by the progressive loss of dopaminergic (DA) neurons in the substantia nigra pars compacta (SNpc) and a reduction in striatal dopamine. In PD, the loss of DA neurons in the SNpc leads to impaired information processing in the basal ganglia [6,7,8]. The main symptoms are rigidity, poor movement, tremors and postural instability [9,10]. Current therapies centre on the oral administration of l-dopa and dopamine receptor agonists, and on deep-brain stimulation in the sub-thalamic nucleus [10]. Although the pharmacological treatment is effective for some symptoms, it has some limitations because its effectiveness decreases over time and side effects develop [11,12]. Thus, an alternative approach for restoration of the damaged DA system is transplantation of DA-synthesizing cells. Human stem cells may provide sources of cells for use in the treatment of PD.

Studies show that stem cells overexpressing neurotrophic factors are able to induce neuroprotective and neuroregenerative effects after grafting in animal models [13,14]. Recently, continuously dividing immortalized cell lines of neural stem cells (NSCs) have been generated by introduction of oncogenes, and these immortalized NSC lines have advantages for basic studies of neural development and cell replacement therapy or gene therapy studies. To be clinically competitive, a stem-cell-based therapy must lead to long-lasting, significant improvement in mobility, ameliorate currently intractable symptoms, or counteract disease progression. Clinical trials of the transplantation of human fetal DA neurons have shown that cell replacement can produce major, long-lasting improvement in some patients [15,16,17]. To make stem-cell therapy work for PD, dopaminergic neurons with the characteristics of substantia nigra neurons must be produced in large numbers [18]. 

For DA neurons generated from human ESCs and NSCs, survival after transplantation in animal models has been poor and needs to be markedly increased before clinical application will be possible [19]. One potential approach to prevent the death of existing neurons could be to transplant human stem cells engineered to express neuroprotective molecules such as glial-cell-line-derived neurotrophic factor (GDNF) [20]. Another study has demonstrated that retinoic acid treatment and transplanting ESCs to the lessioned brain can lead to the generation of putative DA neurons and functional recovery in Parkinsonian rat model [21]. Meanwhile, a study showed that L1-overexpressing stem cell-derived neural aggregates could enhance survival and migration of transplanted cells, differentiation into DA neurons, survival of endogenous DA neurons, and functional recovery after syngeneic transplantation in the 1-methyl-4-phenyl-1,2,3,6-tetrahydropyridine mouse model of PD [22]. 

In addition, the effects of growth factors on embryonic DA neuron grafts were recently studied [23,24]. The data suggested that long-term and continuous neurotrophic factors support provided by Zuckerkandl’s organ to the transplanted NSC derived DA neurons, helped in their better survival, axonal arborization and integration with host cells, leading to long-term functional restoration in the rat model of PD. For these reasons, it is scientifically and medically significant to test major refinements of cell therapies for Parkinson’s disease.

### 2.2. Stem cells and Huntington's disease

Huntington’s disease is a fatal, intractable disorder that is caused by polyglutamate expansions in the huntingtin protein [25,26,27,28,29]. Neuronal dysfunction and degeneration contribute to the progressive physiological, motor, cognitive and emotional disturbances characteristic of HD. One strategy for therapy of HD is to enhance neurogenesis, and the recent treatment of HD has centered on cell therapy strategies to protect vulnerable neuronal cell populations or to replace dysfunctional or dying cells [30,31,32,33,34]. Stem-cell therapy aims to restore or preserve brain function by replacing and protecting striatal neurons. At this time, using stem cells for the delivery of trophic factors and neuroprotection to prevent disease progression seems a more achievable clinical goal in HD than neuronal replacement. 

Most of the recent work in stem cell therapy has been conducted in animal models of HD. The study showed that cell replacement using grafts of fetal striatal neurons promoted functional recovery, and some evidence from clinical trials indicates that this could also occur in patients [35]. The study demonstrated that hMSCs could affect endogenous cells in the striatum of HD mice by increasing cell proliferation, neuronal differentiation, and neuronal cell recruitment, and implantation of hMSCs could prevent striatal atrophy associated with HD [36]. Moreover, human NSCs implanted into the brains of rats were recently found to reduce motor impairments in experimental HD through trophic mechanisms [37]. Human NSCs appear to behave similarly to murine-derived NSCs in rodent models of HD. Intravenously transplanted human NSCs migrate to the striatum, reduce striatal atrophy, and contribute to functional improvement in a rodent lesion model of HD [38,39].

However, protocols and procedures developed from trials of fetal-derived cell transplantation in humans with HD lay the ground-work to move stem cell therapy into the clinic [40]. One of the first challenges to stem cell therapy in HD is to determine which source of stem cells is most efficacious, and many sources have been examined. In addition to human ESCs, stem cells derived from mesenchyme in adults have been investigated as a readily available source of stem cells in HD. Following transplantation into a mouse model of HD, murine ESC-derived NPCs, genetically modified to promote neuronal differentiation, formed GABAergic neurons with appropriate out-growth [41]. Besides, already the method of graft preparation of NSCs for transplantation, as well as the timing of the transplantation procedure strongly could affect the survival of the donor cells when grafted into the quinolinic acid (QA)-lesioned striatum of adult mice [41]. Furthermore, the studies clearly pointed to the fact that immune responses in the CNS are complex, but cannot be ignored when it comes to repair strategies involving cellular transplants. New experimental studies are definitely needed in order to better comprehend and delineate the immunological privilege of the brain [42,43,44]. The results of those experimental studies will allow defining more precisely the extent to which immunosuppressive regimens can be adapted to the specific case of intracerebral transplants [45,46,47].

The grafts were also functionally beneficial given that the animals showed improvement in rotational behavior. In HD, mitochondrial defects are thought to play a significant part in the pathomechanism for cell death in HD pathology. Administration of 3NP, which inhibits mitochondrial succinate dehydrogenase (SDH), can induce the behavioral and anatomical features of HD in rodents and primates. Moreover, data demonstrated that stem cell factor (SCF), produced in situ in the lesioned striatum, was an important factor in promoting the engraftment of stem cells within the lesioned brain which could be able to activate the SCF receptor c-kit and its signaling pathway and to promote the migration and proliferation of mesenchymal and neural stem cells *in vitro* [48,49,50].

Taken together, neural implantation of stem cells may be of benefit in HD but a number of parameters of dose, treatment schedule, and route of administration need to be optimized.

### 2.3. Stem cells and Alzheimer's disease

Alzheimer’s disease (AD) is characterized by neuronal and synaptic loss throughout the brain, involving the basal forebrain cholinergic system, amygdala, hippocampus and several cortical areas [33,51,52,53,54]. Although in AD massive neuronal loss only occurs in very few brain structures, such as the hippocmpal CA1 and CA2 regions, the entorhinal cortex and the locus coeruleus, large parts of the brain are affected by pathological alterations and decreased neuronal metabolism [55,56,57]. Current therapies, such as treatment with acetylcholinesterase inhibitors to enhance cholinergic function, provide only partial and temporary alleviation of symptoms [34]. The pathological changes seen in AD offer an extremely problematic situation for cell replacement. The data show that neural stem cells release diffusible factors that may improve the survival of aged and degenerating neurons in human brains [58]. 

Alzheimer’s disease amyloid precursor protein [13] has been implicated in many neurobiologic processes, but supporting evidence remains indirect [59,60,61,62]. Studies are confounded by the existence of two partially redundant APP homologues, APLP1 and APLP2 [63]. The stem cell culture provides an excellent tool to circumvent the problem of lack of viability of APP/APLP triple knockout mice and would help to explore the function of this intriguing protein further *in vitro* and *in vivo*. A morphological abnormality of neurally-differentiated NSCs has also been described, which we too have seen in NSCs transfected with wild-type APP [64]. Although it remains unclear as to whether or not adult neurogenesis is essential for normal cognitive function in aging [65], it is tempting to speculate that the altered APP metabolism that impairs proper NSCs migration and differentiation could be a part of the pathological process of AD, particularly since aged transgenic APP mice exhibit neocortical neuronal loss [66,67]. Furthermore, although the rate of neuroregeneration in the adult brain may be minimal, it may be that, in the long run, such a deficit significantly reduces normal brain function. In addition to these drawbacks, the use of transplantation therapy for AD with NSCs may not be effective in an environment where APP metabolism is altered and might lead to excessive gliogenesis. They considered the regulation of APP processing to develop effective NSC transplantation therapy for AD patients [68].

Because stem cells can be genetically modified to carry new genes and have high migratory capacity after brain transplantation, they could be used in place of fibroblasts that are known for their immobility following transplantation for delivery of nerve growth factor (NGF) to prevent degeneration of basal forebrain cholinergic neurons [69,70]. However, because stem cells can be genetically modified and have migratory capacity after transplantation, they could be used for the delivery of factors that can modify the course of the disease [71,72]. In support of this approach, basal forebrain grafts of fibroblasts that produce NGF, which counteracts cholinergic neuronal death, stimulates cell function and improves memory in animal models, have been of some benefit in patients with AD [73].

### 2.4. Stem cells and Amyotrophic Lateral Sclerosis

Amyotrophic Lateral Sclerosis is an adult-onset neurodegenerative disorder characterized by degeneration and loss of motor neurons in the cerebral cortex, brainstem and spinal cord, leading to fatal paralysis [74,75]. A stem-cell therapy could restore or preserve the function of both upper and lower motor neurons, and new neurons could become integrated into existing neural circuitries [76,77].

Recent studies have indicated that it is possible to generate motor neurons in culture from stem cells that include ESCs and NSCs. Mouse ESC-derived motor neurons transplanted into motor neuron-injured rat spinal cord survived and extended axons into ventral root, and human ESCs transplanted into cerebrospinal fluid of rats with motor neuron injury migrated into spinal cord and led to improved motor function [78,79,80]. Reports have also shown that it is possible to generate lower motor neurons *in vitro* from stem cells of various sources, including ESCs and those from the fetal CNS [81]. Mouse ES-cell-derived motor neurons establish functional synapses with muscle fibres *in vitro*, and extend axons to ventral roots after transplantation into adult rats, but whether these neurons can integrate into existing neural circuitries and restore motor function has not been established. Whereas neuronal replacement in ALS patients seems a distant goal, using stem cells to prevent motor neurons from dying is a more realistic and shorter-term clinical approach. This prospect is supported by studies showing that human embryonic germ cells delivered into the cerebrospinal fluid of rats with motor neuron injury can migrate into the spinal cord and induce motor recovery, probably through neuroprotection [82,83,84]. The efficacy of this approach could be improved by genetically modifying the stem cells to secrete molecules that promote motor neuron survival. 

It is unrealistic to expect that the transplantation of stem cells or stem cell-derived motor neurons in ALS patients in a clinical setting replaces lost neurons, integrates into existing neural circuitry, and restores motor function. Rather, preventing cell death in host motor neurons via provision of neurotrophic factors by transplanted stem cells or stem cell-derived motor neurons is a more realistic and achievable approach [85,86,87]. For instance, a recent study showed that human cortical progenitors that were engineered to express GDNF survived implantation into the spinal cords of ALS rats and released the neurotrophic factor [88]. Furthermore the studies indicated that ESC-derived cell populations can be directed to express disease-relevant genes and to display characteristics of the disease-specific cell type. These genetically manipulated ESC-derived motor neurons can facilitate and advance the study of disease-specific cellular pathways, and serve as a model system to test new therapeutic approaches [23,89,90,91].

The recent breakthroughs in stem cell research might nevertheless provide possibilities for neural implantation and cell replacement therapy for patients with ALS. Kim *et al.* showed that intrathecal injection with an optimized cell number could be a potential route for stem cell therapy in ALS patients. They suggested that at this dose of 1 × 10^6^, the average number of motor neurons was significantly higher than others, and most injected hMSCs distributed in the ventricular systemand subarachnoid space [92,93]. Additionally, the studies suggested that successful stem cell therapy for ALS likely would require that the cells be combined with other drugs or treatments, such as antioxidants and/or trophic molecules. Many exciting studies are taking this direction; both *in vitro* and *in vivo* studies have shown generation of motor neurons from human ESCs and functional engraftment of these motor neurons after transplantation into the developing chick and adult rodent spinal cord with axonal outgrowth toward muscle [88,94,95,96,97]. Recently, a Phase I clinical trial confirmed that MSCs transplantation into the spinal cord of ALS patients is safe and that MSCs might have a clinical use for future ALS cell based clinical trials [88]. 

### 2.5. Stems cell and stroke

Once stroke damage has maximized, little can be done to recover premorbid function. In addition to therapies aimed at improving cerebral blood flow, there has been increasing emphasis on neuroprotective strategies. Recent attention has focused on restoring brain function through cell transplantation [98,99,100,101,102,103]. A variety of cell types have been tried for restoration of brain function after stroke, mostly in rodent models. The technical and ethical difficulties associated with these cells promoted a search for alternatives. Autologous somatic stem cells are a very attractive source, and there are no ethical concerns and graft rejection is not an issue. However, it is not clear that somatic cells can are plastic enough and can be safely induced to a neural fate. 

A recent study has reported that, in humans with ischemic infarct, intracerebral implantation of human teratocarcinoma NT2-derived neurons has resulted in functional improvement As a first step towards this goal, human fetal NSCs were transplanted into the brains of stroke-damaged rats, resulting in the migration of new neurons towards the ischaemic lesion [104]. Other studies showed that monkey ES-cell-derived progenitors transplanted into the brains of mice after stroke differentiated into various types of neuron and glial cell, re-established connections with target areas, and led to improved motor function [105,106,107,108,109]. In the present report, the possible therapeutic strategy of the stem cell transplantation for the stroke is discussed. Transplanted human NSCs migrated to the lesion site and differentiated into neurons and astrocytes, and three to twelve weeks post-transplantation, a functional improvement was observed in the tranplanted animals compared with nongrafted controls on rotarod and turning-in-an-alley tests. For several months after a stroke, NS cells can generate new striatal neurons that migrate to the site of damage. It is now important to establish whether endogenous neurogenesis can contribute to functional recovery after stroke, and whether it occurs in humans [110].

The therapeutic efficacy of such strategies could be improved further by genetically modifying the stem cells, for example, by overexpressing an anti-apoptotic gene. Interestingly, the stroke-damaged adult rodent brain has some capacity for neuronal replacement from its own NSCs [111]. The distinct population of progenitor cells in the bone marrow is thought to retain the potential for both neural production and differentiation, and may contribute to a therapeutic strategy for stroke. And, because the regeneration of cortical neurons will be the basis for functional improvement in most stroke-damaged brains, it is also needed to know whether the adult brain’s own NS cells can be triggered to produce cortical neurons [112]. For maximal functional recovery, however, regenerative therapy may need to follow combinatorial approaches, which may include cell replacement, trophic support, protection from oxidative stress, and the neutralization of the growth-inhibitory components for endogenous neuronal stem cells.

### 2.6. Stem cells and spinal cord injury

Spinal cord injury (SCI) invariably results in the loss of neurons and axonal degeneration at the lesion site, leading to permanent paralysis and loss of sensation below the site of the injury. It is interrupt ascending and descending axonal pathways, and cause a loss of neurons and glia, inflammation and demyelination [113]. 

Preclinical studies have been performed on rats with a spinal cord injury and have shown that transplanted MSCs in the injured spinal cord survive, migrate into the host tissue and lead to axonal regeneration and motor function recovery [114]. Dasari *et al.* showed that expression of caspase-3 on both neurons and oligodendrocytes after SCI was significantly down-regulated by MSCs [114,115]. And treatment with MSCs had a positive effect on behavioral outcome and histopathological assessment after SCI. There is no cure, and the most common current treatment high-dose methylprednisolone is of questionable value. The transplantation of stem cells into injured spinal cord can lead to functional benefits, mainly through trophic factor secretion or the remyelination of spared axons. A recent study showed that human NS cells implanted into damaged mouse spinal cord generated new neurons and oligodendrocytes, leading to locomotor recovery [116]. 

In addition, MSCs are attractive targets for *ex vivo* cell and gene therapy. Ronsyn *et al.* investigated the feasibility of a plasmid-based strategy for genetic modification of human MSCs with enhanced green fluorescent protein (EGFP) and neurotrophin (NT) [117]. Bakshi *et al.* found that MSCs delivered by lumbar puncture (LP) reached the contused spinal cord tissues and exerted a significant beneficial effect by reducing cyst and injury size [118]. Astrocytic differentiation and aberrant axonal sprouting after NSCs implantation into injured rat spinal cord can cause hypersensitivity to stimuli [119]. With regard to the yet controversial immunological status of stem cells, it is important to predict strategies to overcome the potential immunoincompatibility. To reach this aim, two main possibilities can be foreseen. Banking of hESCs including only 150 donors with unique blood groups could provide a beneficial HLA matching for most potential patients. If confirmed, such a bank could be generated under GMP conditions and would avoid the need of somatic cell nuclear transfer to customize hESCs, a yet not successful approach in human beings [120]. Although chimerism between ESCs and recipients has been reported, another strategy to confer some immune tolerance to HES would be to generate tolerogenic hematopoietic cells derived from them. Together, these strategies demonstrate the possibilities to overcome the immunologic barrier [121]. 

## 3. Conclusions

This review has discussed the major issues associated with stem cell therapy by transplantation for neurodegenerative diseases. Stem cells from a variety of sources have shown effectiveness in improving motor function after neurodegenerative diseases in animal experiments and clinical trails. Cell therapies in neurodegenerative disease are intended to protect neuronal populations susceptible to disease and replace dysfunctional or dying neurons. The use of both stem cell and growth factor–based therapies, although in its early stages, appears likely to contribute to future clinical strategies, including, but not limited to, neuroprotective and neuron replacement approaches. However, factors that control the differentiation, survival, and maturation of stem cells in the context of a host degenerative brain must be more thoroughly understood before stem cell therapy will prove to be a robust and safe strategy that can be transferred to the clinic. Furthermore, long-term and large scale multicenter clinical studies are required to determine further the precise therapeutic effect of stem cell transplantation.
